# The importance of peripheral populations in the face of novel environmental change

**DOI:** 10.1098/rspb.2024.2331

**Published:** 2025-01-08

**Authors:** Samantha Hoff, Joseph R. Hoyt, Kate E. Langwig, Luanne Johnson, Elizabeth Olson, Danielle O’Dell, Casey J. Pendergast, Carl J. Herzog, Katy L. Parise, Jeffrey T. Foster, Wendy C. Turner

**Affiliations:** ^1^ Department of Biological Sciences, University at Albany, 1400 Washington Avenue, Albany, NY 12222, USA; ^2^ New York State Department of Environmental Conservation, 625 Broadway, Albany, NY 12223, USA; ^3^ Department of Biological Sciences, Virginia Polytechnic Institute and State University, Blacksburg, VA 24061, USA; ^4^ BiodiversityWorks, 455 State Road PMB#179, Vineyard Haven, MA 02568, USA; ^5^ Nantucket Conservation Foundation, 118 Cliff Road, Nantucket, MA 02554, USA; ^6^ Pathogen and Microbiome Institute, Northern Arizona University, Flagstaff, AZ 86011, USA; ^7^ Department of Forest and Wildlife Ecology, US Geological Survey, Wisconsin Cooperative Wildlife Research Unit, University of Wisconsin-Madison, 1630 Linden Drive, Madison, WI 53706, USA

**Keywords:** peripheral populations, refugia from disease, host persistence, host–pathogen interactions, white-nose snydrome, northern myotis

## Abstract

Anthropogenically driven environmental change has imposed substantial threats on biodiversity, including the emergence of infectious diseases that have resulted in declines of wildlife globally. In response to pathogen invasion, maintaining diversity within host populations across heterogenous environments is essential to facilitating species persistence. White-nose syndrome is an emerging fungal pathogen that has caused mass mortalities of hibernating bats across North America. However, in the northeast, peripheral island populations of the endangered northern myotis (*Myotis septentrionalis*) appear to be persisting despite infection while mainland populations in the core of the species range have experienced sharp declines. Thus, this study investigated host and environmental factors that may contribute to divergent population responses. We compared patterns of pathogen exposure and infection intensity between populations and documented the environmental conditions and host activity patterns that may promote survival despite disease invasion. For island populations, we found lower prevalence and less severe infections, possibly due to a shorter hibernation duration compared to the mainland, which may reduce the time for disease progression. The coastal region of the northern myotis range may serve as habitat refugia that enables this species to persist despite pathogen exposure; however, conservation efforts could be critical to supporting species survival in the long term.

## 1. Introduction

Anthropogenic processes have caused rapid environmental change that poses a significant threat to global biodiversity. These processes include habitat loss and fragmentation, spread of invasive species, global climate change and emergence of infectious diseases [1,2] and the effects can scale from individuals to ecosystems [3,4]. The current pace of change is unprecedented and may occur in unpredictable ways, raising important questions about whether species will have the ability to respond evolutionarily or ecologically at rates necessary to survive [4]. Given the complex nature of these processes, understanding the functional responses of populations to variable environmental conditions is critical to informing species conservation and management.

The conservation value of peripheral populations, or those that occur at range edges, and the role they play in species persistence is underappreciated [5]. Peripheral populations have at times been considered demographic sinks due to their small size and isolation [6], factors that typically interact to increase the likelihood of local extirpation. Occupancy may be limited at the periphery due to varying biotic and abiotic factors across a species range [7], and thus peripheral populations are often considered to occur in suboptimal or marginal habitat resulting from extreme or variable environments [8,9]. However, these same factors that provide variation can subsequently serve as refugia under novel stressors such as the emergence of infectious disease. Populations may balance the costly adaptations needed to survive in peripheral habitats by receiving fitness benefits from reductions in frequency and/or intensity of negative biotic interactions (i.e. pathogen refuge hypothesis [10]), which allows individuals to reduce or completely avoid infection as these habitats act as refuge from parasites and disease [11,12]. Given the variability of factors that limit a species range (e.g. spatial variations in the environment at northern and southern range limits [13]), we would not expect all peripheral populations to serve as refugia. The value of peripheral populations may depend on the specific environmental conditions and species interactions of interest, as these factors have the potential to affect host population size and mediate disease risk.

Identifying the factors that moderate the relationship between hosts, pathogens and their environments is essential for a more comprehensive understanding of disease dynamics within core versus peripheral populations and to develop effective management actions. Recent studies focusing on peripheral populations have explored the species traits and environmental conditions that may mediate the effects of climate change-driven range shifts [5,9]. However, the potential for these populations to play a role in promoting species persistence against infectious disease is relatively unexplored. Host population-level responses to emerging infectious diseases can vary within a single species and across spatial scales as some populations stabilize and persist at low densities following the initial epidemic, although the mechanisms leading to these outcomes are not well understood [14–16]. The variation in extinction risk between core and peripheral populations may be linked to potential drivers of host–pathogen coexistence, including differing environmental conditions and species communities. The presence of environmental refugia may facilitate species persistence where local abiotic conditions and corresponding behavioural differences alter host–pathogen dynamics or the effects of disease on individuals [17,18] by making pathogen establishment or survival on hosts less likely, even for highly susceptible species [19,20]. If these factors support greater survival and reproduction of individuals within the periphery, they may play a key role in supporting long-term species persistence in response to novel stressors.

We investigated how peripheral versus core bat populations are affected by the invasion of a fungal pathogen. White-nose syndrome (WNS) has emerged as a multi-host infectious disease of North American bats and is caused by the invasive fungal pathogen *Pseudogymnoascus destructans* [21]. Since its arrival in 2006, the pathogen has rapidly spread across the continent, resulting in mass mortality events and regional extirpations of once common bat species [22,23]. The disease is characterized by cutaneous fungal infection that disrupts critical physiological processes during hibernation and often leads to mortality [24,25]. Individuals able to survive the winter emerge from hibernation in spring and clear infection, although downstream fitness costs are unknown [26]. WNS does not impact all bat species equally, and factors such as roosting behaviour or environmental conditions of hibernacula can positively or negatively affect survival rates, resulting in stabilization for some populations despite infection prevalence remaining high while other populations continue to decline or become locally extirpated [15,16,27].

The northern myotis (*Myotis septentrionalis*) is one of the species most affected by WNS, with colony count declines exceeding 95% and complete extirpation occurring from many hibernacula in the northeast and Midwest USA [21,22]. WNS has currently been confirmed in over 79% of this species range, and as the pathogen spreads, it continues to cause rapid extirpation from hibernacula within 3 years of arrival, constituting the most severe and pervasive threat for this species [22,28]. The northern myotis was recently reclassified as federally endangered in the United States [29], reflecting the dire situation faced by this species. One source of hope lies at the edge of the species range where recent evidence suggests populations are persisting in locations that were either understudied in pre-WNS years [30] or previously thought to be outside of the species range [31–34]. Some of these locations along the Atlantic coast remain apparently unaffected by WNS as *P. destructans* has yet to be detected [35,36], while others have documented fungal presence, but no mass mortalities. Peripheral populations of northern myotis may be dictated by different environmental processes (perhaps limited abundance at the northern edge due to climate and tree community changes or increased competition due to a species rich bat community in the south [13]) and thus may respond differently to stressors. For this study, we focus specifically on the factors influencing the persistence of peripheral populations along coastal islands in the northeastern United States.

We conducted a comparative study of northern myotis populations to explore the mechanisms that may be supporting species survival. Here, the core of the species range is represented by mainland populations and the periphery is represented by the coastal island populations of Long Island, New York, and Martha’s Vineyard and Nantucket, Massachusetts. We predicted that the coastal region is acting as refugia to support population persistence due to diverging environmental conditions that vary between the core versus periphery and ultimately shape disease dynamics (electronic supplementary material, table S1). First, we presented mainland colony declines from hibernacula across the species range and we compared capture probabilities of island and mainland bats to assess the severity of population decline in post-WNS years. We quantified seasonal patterns of *P. destructans* prevalence and infection intensity between the islands and mainland, predicting that both metrics would be lower at the end of hibernation for island populations. The duration of hibernation is a vital determinant to the survival of WNS-affected bats [21,37]; therefore we modelled the predicted length of hibernation for the islands and mainland in relation to local climate conditions and measured seasonal bat activity on the islands to document the periods of hibernation entry and emergence. In addition, hibernacula microclimates influence the growth rate of *P. destructans* and the stability of the environmental reservoir [21,38,39], factors that contribute to the degree of infection and likelihood of exposure for hibernating bats. Therefore, we compared the temperature and humidity of island and mainland hibernacula over the winter season to assess if microclimates differ by location. Severe WNS infections typically result in poor body condition at the end of hibernation [26], and thus we compared spring body weights of island bats to evaluate differences in body condition between pre- and post-WNS years and assessed the degree of post-WNS wing damage due to prior infection. By disentangling the factors contributing to northern myotis persistence, we may be able to develop more informed conservation strategies to support these populations, and potentially those remaining across the range of this species, in the long term.

## 2. Material and methods

### (a) Data collection and processing

Island bats were captured using mist-nets to sample individuals prior to entering hibernation (14 October–11 November) and following emergence during spring (28 March–31 May) between 2017 and 2021 across 26 sites (see electronic supplementary material, figure S12 and table S2, for study area description). All individuals were sexed and received a unique aluminium band to document recaptures. For bats captured during spring, we recorded three disease severity metrics: (i) body condition, measured by body mass (g); (ii) a standardized 4-point wing-damage index (WDI) [40] to assess the incidence and severity of wing damage after expected exposure to WNS; and (iii) an ultraviolet light (UV) score, measured as the area of the wing with orange fluorescence, to document the degree of infection (0, none; 1, 1–10%; 2, 10−50%; 3, ≥50% of wing area [41]). We collected cutaneous swab samples from each individual using a standardized swabbing protocol described by Langwig *et al.* [42]. Mainland bats were sampled during autumn swarm or hibernation (10 August–27 March) between 2011 and 2021 at 30 cave or mine hibernacula across five states (electronic supplementary material, figure S1 and table S2) [43]. Additionally, we collected between 5 and 31 (*n* = 51) environmental swabs from four island hibernacula during the autumn swarm through the maternity season based on when we were permitted access by homeowners (see electronic supplementary material for site description). We extracted DNA from bat and environmental swab samples using a blood and tissue kit (Qiagen) following the modifications described by Shuey *et al.* [44]. We tested for the presence and quantity of *P. destructans* using qPCR [45] and used a standard curve to determine fungal load [46]. Capture and handling procedures were approved by the Animal Care and Use Committees of the University at Albany and Virginia Tech.

To measure hibernacula microclimates, we placed temperature and humidity loggers (iButton model DS1923 Hygrochron, ±0.5°C and ±0.6% RH; Maxim Integrated Products, Inc.) in six anthropogenic hibernacula on the islands. Loggers were deployed between mid-November and late April for one winter season per site (either 2018−2019 or 2020−2021 depending on the year of site discovery). We deployed 1−3 loggers within each site at locations where bats had been observed roosting and set loggers to take readings every 2 h to capture daily temperature extremes. We obtained temperature and humidity data from three hibernacula on the mainland of New York that had some of the highest winter colony counts of northern myotis in pre-WNS years; sampling within these sites was conducted over the 2003−2004 hibernation season with 3−7 temperature and relative humidity loggers (HOBO H08 Pro Series; Onset Computer Corporation) deployed in each site to take a reading every 3 h.

### (b) Statistical analysis

To document population declines at mainland hibernacula, we estimated the population growth rate, λ, for colonies with counts before and after WNS detection at 74 sites across 8 states (these data are from state government reports or publicly available from the North American Bat Monitoring Program [47]). We used the single most recent pre-WNS census as a proxy for colony size prior to the arrival of WNS, which was an average of 2.1 years (range 1−9) before WNS detection, and all sites had at least two surveys post-WNS detection (range 2−4). Because there are no comparable data of hibernacula colony counts available for the islands, we modelled the probability of capturing a northern myotis in a nightly mist-net survey (9 April–1 December) across years since WNS arrival (YSW) for the islands (YSW range 0−9) and the mainland of New York and Wisconsin (YSW range 0−10). We fitted a generalized linear binomial model with the interaction of YSW and location (island versus mainland) and included an offset of northern myotis captured per net night to account for differences in effort. For island bats captured during spring, we analysed three metrics to assess disease severity among these populations. We used linear regression on body mass measurements to assess the difference in body condition between bats during pre- and post-WNS years. To test for differences in the severity of tissue invasion, we used a Fisher’s exact test to compare the proportion of bats assigned each WDI and UV score.

For the following models exploring prevalence and infection intensity, we first converted the sampling date to a modified time variable (time) beginning the first day that autumn sampling occurred and expressed in units of partial months. Because no *P. destructans* surveillance efforts occurred on the islands prior to our study, we used the winter of the first documented WNS mortality on Long Island (2010/2011) and the first *P. destructans* detection on Nantucket (2017/2018) as the year of WNS arrival. For Martha’s Vineyard, we used the year of the first *P. destructans* detection in the closest county (2013) as the year of WNS arrival. We then compared seasonal prevalence among the islands by fitting a generalized linear binomial model with time and island as fixed effects and survey site as a random effect. The results indicated a significant difference in pathogen prevalence for Nantucket (3%, 2/79 positive samples) compared to Long Island and Martha’s Vineyard, and thus we assigned Nantucket disease data to the invasion phase (years 0−3) and Long Island and Martha’s Vineyard data to the post-invasion phase (years 4+) for subsequent analysis. To examine differences in prevalence between island and mainland populations over time, we fitted two binomial models: (i) time and the interaction of phase (invasion versus post-invasion, all island invasion data originating from Nantucket) with location (island versus mainland) as fixed effects and winter sampling year as a random effect to compare transmission between locations and WNS phase over time and (ii) location and year since WNS arrival (YSW) as fixed effects and survey site as a random effect to estimate the change in prevalence since WNS arrival. To compare infection intensity, as measured by qPCR analysis of fungal loads, we modelled seasonal changes of fungal loads between island and mainland populations over the hibernation season with the interaction of season (autumn versus spring) and location as fixed effects, winter sampling year as a random effect and Nantucket data removed (due to low sample size of positive bats). We compared environmental samples from one island hibernaculum (*n* = 31) with a binomial generalized linear model to estimate the change in *P. destructans* prevalence across multiple years and seasons and a linear regression model to assess the change in fungal load in the environment from early to late hibernation.

We estimated the length of the hibernation season on the islands with two measures: (i) landscape bat activity and (ii) ambient conditions (minimum nightly temperature). For the first method, we used seasonal bat acoustic data to estimate the length of the hibernation season for each year separately and then calculated the mean range for all survey years combined to provide estimates of hibernation entry and spring emergence dates (see electronic supplementary material, table S3, for acoustic data collection and analysis process). Following the methods of Meyer *et al.* [48], we defined the entry into and exit from hibernation as the days when cumulative activity declined below 5% or 1% of the autumn total, and reached 1% or 5% of the spring total, respectively. We conducted a Kruskall–Wallis test to determine if our estimated durations differed across years.

For the ambient conditions method, we obtained minimum nightly temperatures from the National Climatic Data Center [49] for each island at the closest weather station to six anthropogenic structures used as hibernacula. Additionally, we obtained minimum nightly temperatures from three hibernacula located on the mainland of New York that had some of the highest winter colony counts of northern myotis in pre-WNS years to provide a comparison of hibernation length between the islands and mainland. We then fitted a generalized additive model (GAM) to these data for each location, with date predicting the mean minimum nightly temperature. Prior studies suggest the minimum hibernation duration is represented by the number of days where mean nightly temperatures are below 0°C [50], assuming that freezing temperatures will limit insect availability, and thus influence the hibernation period [50,51]. Therefore, we calculated the days below 0°C for each location based on the model fit to quantify the approximate hibernation length for each location, and to compare with our activity-determined hibernation lengths.

To assess the effects of ambient conditions on seasonal bat presence, we calculated the following measurements [49]: maximum day temperature (°C), average nightly wind speed (mph), total hours of nightly precipitation and moon face illuminated (mfi, %). All continuous predictors were *z*-transformed to allow for comparison of beta coefficients and assessment of effect sizes. Prior to analysis, we transformed the survey date into a day of year variable, with the first survey date (1 September) as day 1 and the last survey date (31 May) as day 274. Nightly counts of northern myotis were converted to a binary variable (detected/not detected) to represent presence/absence and we ran a generalized linear mixed model with a binomial distribution using the package glmmTMB [52]. We included month (treated as a factor) as a random effect to account for temporal autocorrelation and included all predictor variables in one global model. To assess the variance explained by the model (including both fixed and random effects), we calculated a pseudo-*R*^2^ [53].

We recorded the average daily temperature at weather stations nearest to island hibernacula [49] to assess the relationship between internal hibernacula temperature and external ambient temperature. Ambient temperature regimes varied among study areas; therefore, we calculated the difference between the daily average island hibernacula temperature and the daily average island ambient temperature (*T*_diff_) to reduce spatial variation. Positive values indicate that island hibernacula temperatures were higher than ambient temperatures. We then estimated the average *T*_diff_ using a mixed-effects model with month as a fixed effect (treated as a sequential factor) and site as a random effect to explore the relationship between ambient and hibernacula temperatures across the hibernation period. To consider the absolute level of ambient moisture within sites, we converted relative humidity to vapour pressure deficit (VPD) by using the daily hibernacula temperature to calculate the saturated vapour pressure, and then multiplied the saturated vapour pressure with scaled relative humidity values to get VPD (kPa). We then compared temperature and VPD between all island and mainland hibernacula with a Kruskal–Wallis test as our data violated assumptions of normality and homogeneity of variances. To assess the variation within sites, we calculated coefficients of variation for each individual site and for grouped island or mainland sites (electronic supplementary material, table S4). We fitted mixed-effects models for temperature and VPD with either site (hibernacula) or location (island or mainland) as categorical fixed effects and month as a random effect to account for variation within sites at the beginning and end of hibernation. All analyses were run in R (v. 4.0.4 [54]), and a table detailing analyses, figures and results is provided in the electronic supplementary material (table S5), which indicates where the results can be found (e.g. electronic supplementary material, table, figure legend or article text), the parameters of the model, and the main findings.

## 3. Results

### (a) White-nose syndrome dynamics

**Figure 1.**
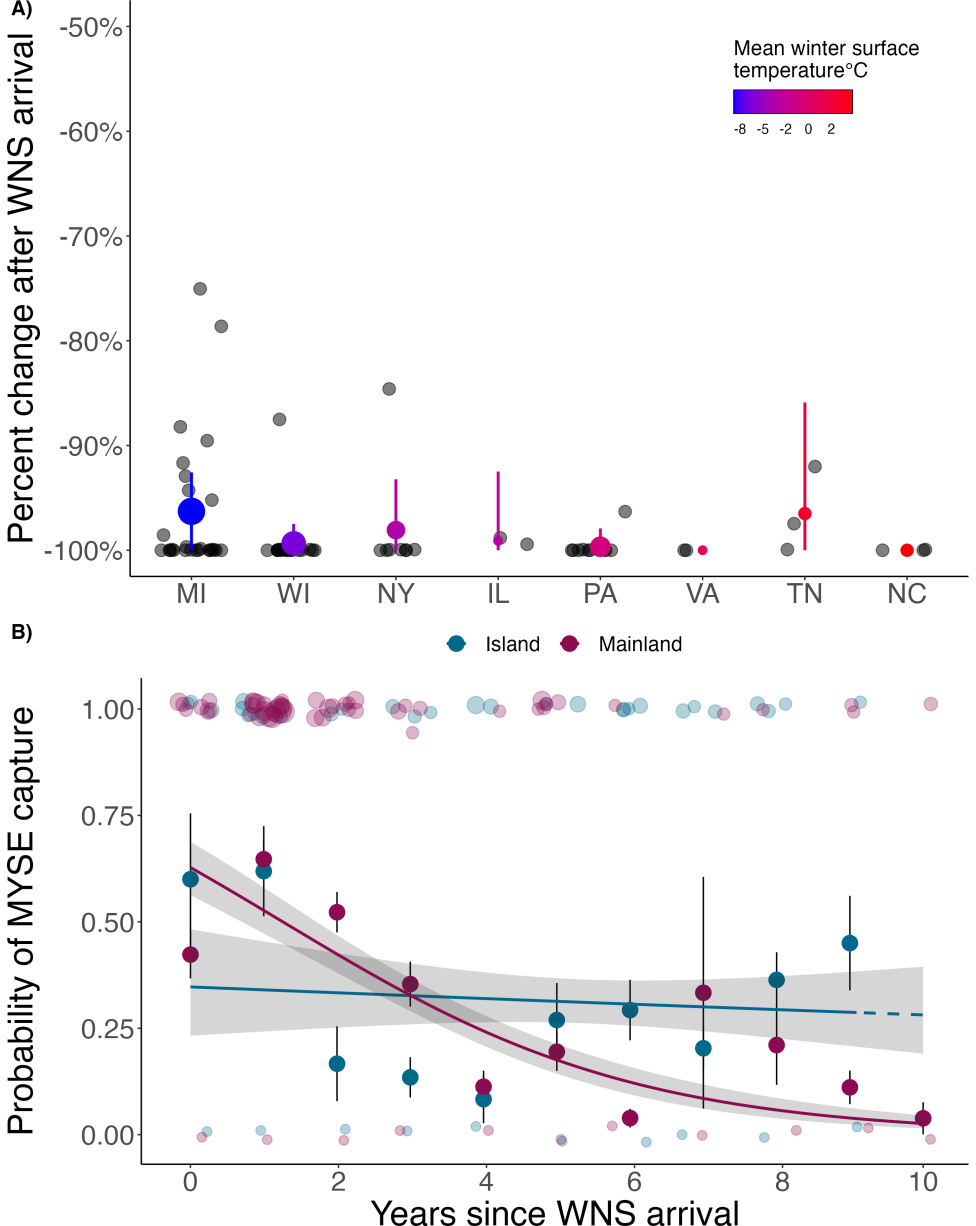
Impacts of WNS on northern myotis (*M. septentrionalis*; MYSE) populations. (A) Percent change in hibernating MYSE populations 1–4 years after *P. destructans* arrival in different US states (shown by two-letter state codes; electronic supplementary material, table S1). Bold points show the mean decline and bars represent ± s.e. States are arranged on the *x*-axis according to latitude, and the colour of each point represents the mean winter surface temperature (°C) at hibernacula locations. Hibernating populations declined an average of 99% within 4 years of *P. destructans* arrival. (B) Probability of capturing a MYSE on one mist-net night over years since WNS arrival (YSW) on the islands of Long Island, New York (LI; 2012–2021), and Martha’s Vineyard (MV; 2014–2021) and Nantucket (N; 2016–2021), Massachusetts, in comparison to nightly capture probabilities on the mainland in upstate New York (NY; 2006–2016) and Wisconsin (WI; 2015–2019). Transparent ribbons represent the 95% confidence intervals, and solid lines show the mean of the posterior distribution. Bold points represent the mean capture probability by YSW, bars indicate ± s.e. and the size of the transparent 0/1 points represent the number of MYSE captured per mist-net night. The dashed line indicates model predictions beyond our sampling time frame. Capture probability on the islands remains at 29% in year 9 while the probability of capture on the mainland drops to 4%.

Within 4 years of WNS arrival, mainland hibernating populations of northern myotis at 74 sites within 8 states declined an average of 99% (range 96−100%; figure 1*a*). While capture probabilities have declined on both the mainland and islands following pathogen arrival, there was a significant difference between locations (reference coeff. (island): −1.39, YSW:mainland coeff.: −0.38 ± 0.06, *z* = −6.18, *p* ≤ 0.001; electronic supplementary material, table S6), such that the probability of capture on the islands remained at 29% each survey night while the probability dropped to 4% on the mainland by year 9 post-WNS (figure 1*b*). During spring mist-net surveys on the islands, our recapture rate was 10% (*n* = 6 of 62 total bats) and included five individuals that tested positive for *P. destructans*, which may represent multi-year survivors despite infection (overall recapture rate across all seasons and survey years was 7% or *n* = 9 of 125 total bats). Comparisons of spring body mass were not significantly different between island bats in pre- and post-WNS years (pre-WNS coeff: −0.009 ± 0.03, *t* = −0.28, *p* = 0.78; electronic supplementary material, figure S2). All spring-captured island bats were assigned the lowest two categories of the WDI (0 or 1; electronic supplementary material, figure S3A), and most bats were assigned a UV score of 0 or 1 (98% of individuals, with 10% or less of the wing area displaying UV fluorescence; electronic supplementary material, figure S3C).

**Figure 2.**
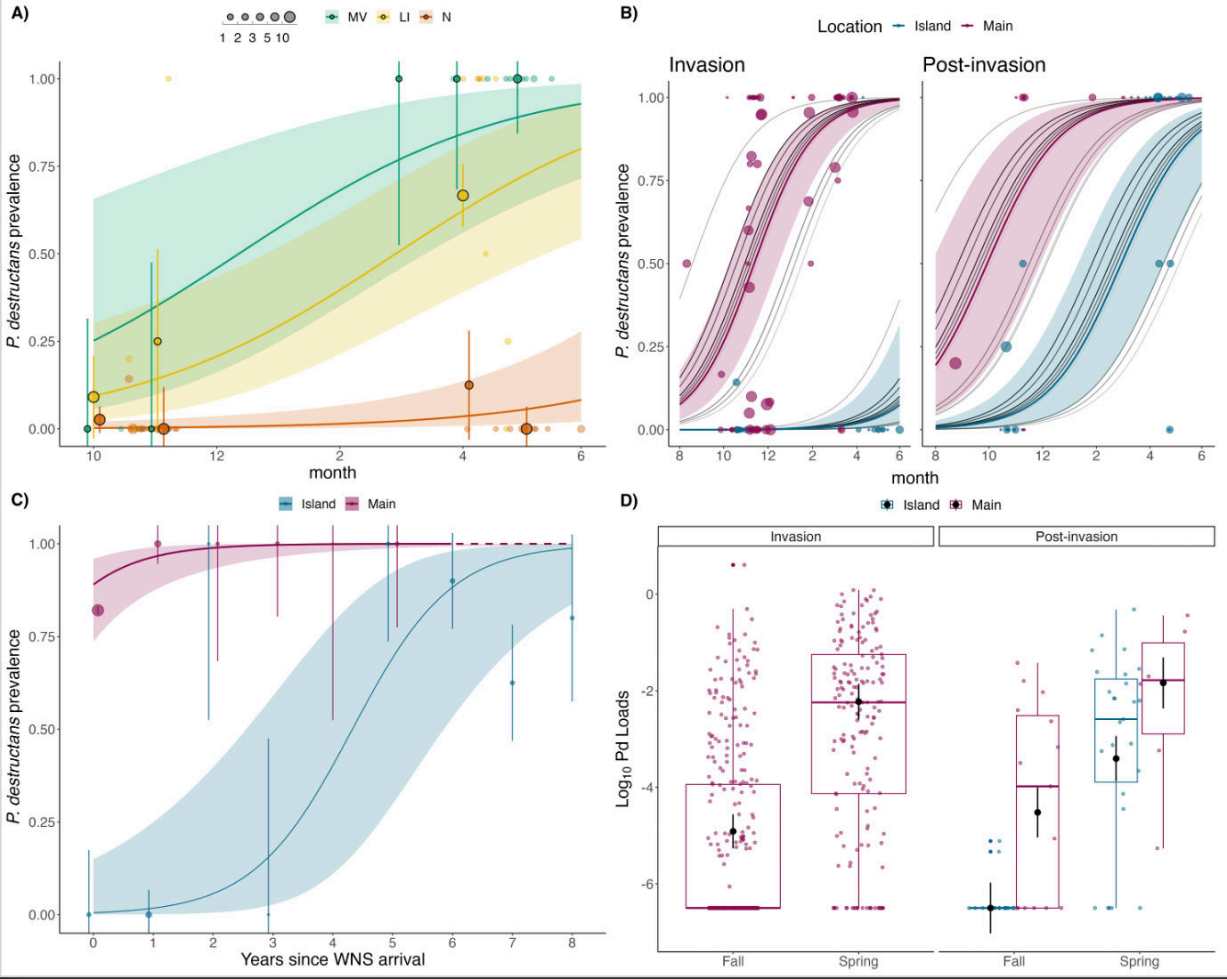
WNS disease dynamics in northern myotis (*M. septentrionalis*) populations. (A) Seasonal prevalence of *P. destructans* among island populations (LI = Long Island, New York, MV = Martha’s Vineyard, Massachusetts, N = Nantucket, Massachusetts) over time (2017–2021, *n* = 125 bats sampled). Bold points show mean prevalence by month, bars represent ± s.e. and size of the points represents sample size for each month or sampling day (transparent points). (B) Seasonal prevalence of *P. destructans* by location (island versus mainland; *n* = 530 bats sampled on the mainland, 2011–2021) during invasion (years 0–3) and post-invasion years (4+). Size of the points represents sample size for each sampling day, and grey lines depict model predictions with increasing darkening for each year of sampling. (C) *P. destructans* prevalence by location across years since WNS (YSW) arrival (spring data only when prevalence is highest). The dashed line indicates model predictions for years where no individuals were available to sample. Bold points show mean prevalence by YSW and bars represent ± s.e. Solid lines in each panel show the mean of the posterior distribution, and transparent ribbons show the 95% confidence intervals. (D) Infection intensity (measured as fungal loads for individual bats, log_10_ ng DNA) between the mainland and islands during invasion and post-invasion years. Black points show mean fungal loads by season and disease phase, bars represent ± s.e. Electronic supplementary material, figure S1, depicts sample sizes for each geographic location; electronic supplementary material, table S1, describes sample sizes for each location, by group (invasion versus post-invasion) and by season; and electronic supplementary material, table S6, displays the model output for each WNS model.

We sampled a total of 125 northern myotis from 26 locations across 5 years from island populations and compared them to 530 bats from 30 locations across 10 years from mainland populations. During our sampling period on the islands (2017−2021), overall prevalence of *P. destructans* was significantly lower on Nantucket (3%, *n* = 2 positives out of 77 samples) compared to Long Island and Martha’s Vineyard, with no significant difference between the latter two islands (40% and 75%, respectively; figure 2*a* and electronic supplementary material, table S7). Prevalence was significantly higher on the mainland in both the invasion (years 0−3) and post-invasion years (4+; figure 2*b* and electronic supplementary material, table S7) and across years since WNS arrival (reference coeff. (island): −3.93, mainland coeff.: 6.01 ± 1.64, *z* = 3.66, *p* ≤ 0.001; figure 2*c* and electronic supplementary material, table S7); however, all island data during the invasion period were collected on Nantucket, and thus it remains unclear whether Long Island and Martha’s Vineyard exhibited a similar prevalence pattern following pathogen arrival. In the post-invasion years, autumn fungal prevalence remained low on Long Island and Martha’s Vineyard (11% of bats infected, *n* = 2 of 19 samples) compared to mainland prevalence (60%, *n* = 9 of 15 samples). At the end of hibernation, prevalence reached 100% on the mainland (*n* = 6) and averaged 81% on Long Island and Martha’s Vineyard (*n* = 27). Infection intensity increased significantly across the hibernation period for both locations (season coeff: 3.34 ± 0.52, *z* = 6.44, *p* < 0.001) and differed between the islands and mainland (reference coeff. (island): −7.14, mainland coeff.: 2.23 ± 0.55, *z* = 4.07, *p* < 0.001, Nantucket data removed; figure 2*d* and electronic supplementary material, table S7), but there was no support for the interaction of season and location. Both prevalence and fungal load of environmental *P. destructans* collected from an island hibernaculum were not significantly different between sampling periods and across the hibernation season (electronic supplementary material, figure S4), and prevalence within this hibernaculum was 52% (*n* = 16 of 31 samples).

### (b) Seasonal activity and hibernation duration

**Figure 3.**
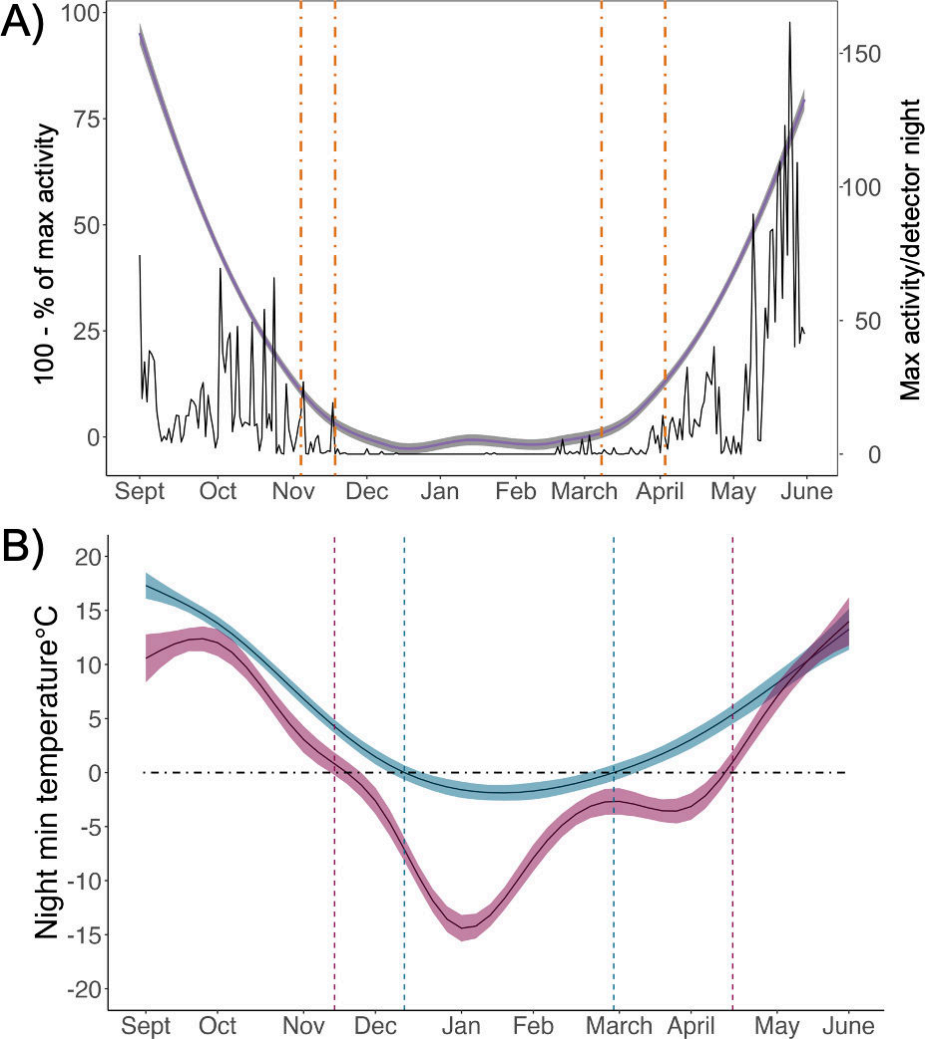
(A) Seasonal activity of northern myotis (*M. septentrionalis*) on the islands of Long Island, New York, and Martha’s Vineyard and Nantucket, Massachusetts. Acoustic data were pooled across all study periods (1 September–31 May 2017–2020) and sampling sites. The solid black line represents the maximum (max) nightly activity/detector night, and the shaded purple line represents 100—the cumulative per cent of maximum nightly activity by season (autumn or spring). Dashed orange lines show the average 5% and 1% activity cutoff dates for the autumn and spring seasons (1% = 20 November–20 March, 5% = 6 November–5 April). Electronic supplementary material, figure S6, depicts the maximum nightly activity by study period. (B) Estimated hibernation duration on the islands and the mainland of New York represented by days below 0°C. Predicted days were calculated from the output of a GAM of average nightly minimum (min) surface temperatures for each location. The black dashed line indicates the 0°C nightly minimum temperature cutoff; the portion of the coloured lines falling below the dashed line indicates the estimated hibernation period (island: 81 days; mainland: 154 days). Average nightly minimum surface temperatures by location are presented in electronic supplementary material, figure S7. Weather data were obtained from the closest weather stations to each hibernacula location within the acoustic sampling period (2017–2020) [49].

Between 1 September and 31 May of 2017−2020 we collected a total of 711 detector nights of acoustic data from the islands and confirmed identification of 14 986 northern myotis calls over 312 detector nights. Northern myotis were detected on 71.2% of autumn nights, 4.5% of winter nights and 60.6% of spring nights across all study years (electronic supplementary materials, table S3 and figures S5 and S6). The maximum day temperature (°C) was the strongest predictor of seasonal northern myotis presence (*β* = 7.44, CI = 4.15−13.31; electronic supplementary material, figure S5A,C), with 63% of the variance in presence explained by the global model. Northern myotis were periodically active throughout the winter hibernation period, with detections occurring at temperatures as low as −2.2°C (*x̅* = 5.5 ± 3.7 s.d.) and on days when the maximum temperature ranged from 1.7 to 13.8°C (electronic supplementary material, figure S4B). The 1% and 5% cutoffs in seasonal landscape bat activity resulted in an estimated range of hibernation entry between 21 October and 20 November and hibernation emergence between 4 March and 11 April that differed by survey year (*F*_2,117_ = 20.74, *p* < 0.001; figure 3*a*). Given that our acoustic sampling coverage on the islands was not comprehensive (spatially or temporally) and we did not have comparable data from the mainland, we estimated the length of winter for each location based on ambient conditions (the number of days where nightly minimum temperatures are below 0°C). The temperature-based method predicted the hibernation duration to be 81 days on the islands compared to 154 days on the mainland (figure 3*b* and electronic supplementary material, figure S7).

### (c) Hibernacula microclimate

**Figure 4.**
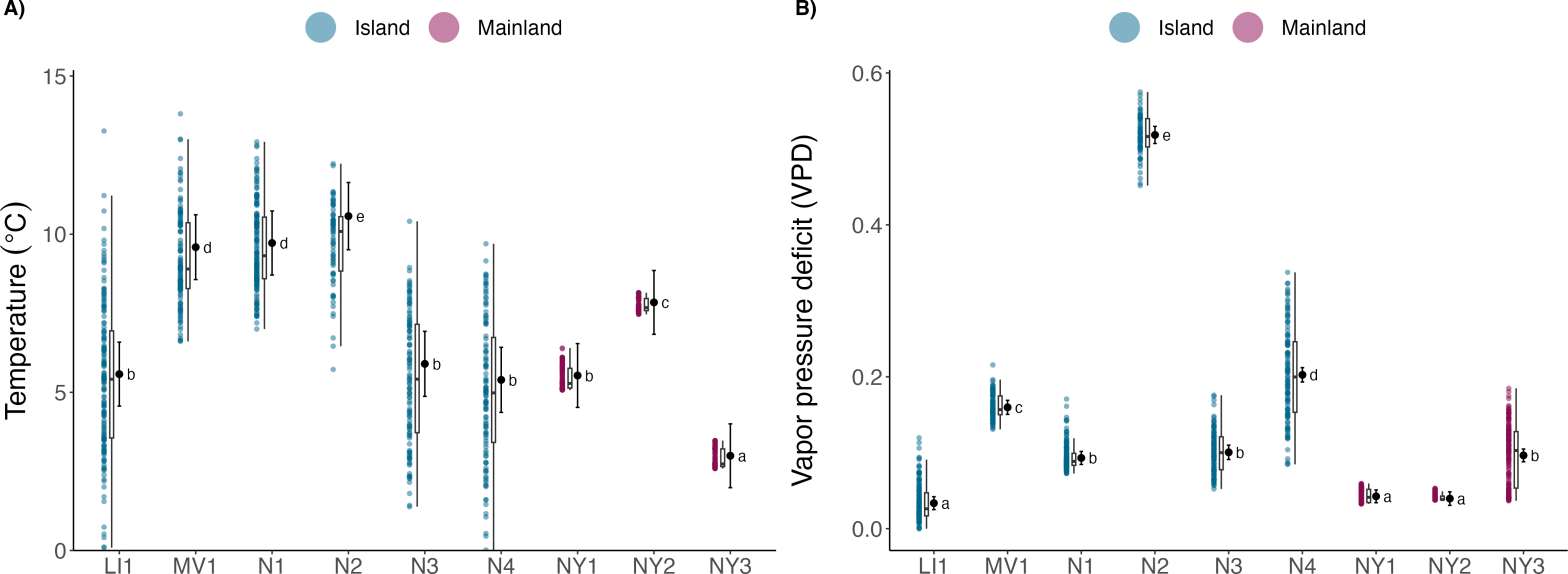
Temperature (A) and VPD () of mainland and island hibernacula sampled over hibernation from November to April. Letters denote the results of pairwise comparisons among all sites. Mean temperatures were greater in island sites (*x̅* = 7.3°C ± 0.3 s.e., range = −1.0 to 13.8) than mainland sites (*x̅* = 5.4°C ± 0.3 s.e., range = 2.59–8.15), and were more variable over the hibernation period (*t* = −2.35, d.f. = 7, *p* = 0.05; mean coeff. of variation: island = 0.29, mainland = 0.06).

Hibernacula temperatures were significantly different between island and mainland hibernacula (mainland location coeff: −2.14 ± 0.15, *t* = −14.7, *p* < 0.001; figure 4*a*). Island sites were warmer on average than mainland sites and more variable over the hibernation period (mean coeff. of variation: island = 0.29, mainland = 0.06; electronic supplementary material, table S4). Average VPD was greater in island sites compared to mainland sites (mainland coeff.: −0.09 ± 0.006, *t* = −13.3, *p* < 0.001; figure 4*b*), suggesting that island sites are on average drier than mainland sites across the hibernation period. *Post hoc* pairwise comparisons showed that temperature and VPD differed among most sites (figure 4*a*,*b*); however, there was no identifiable trend by location or site. Ambient temperatures were more variable than island hibernacula (coeff. of variation: hibernacula = 0.39, ambient = 1.40; electronic supplementary material, figure S8) across the sampling period, and among all island sites, only one hibernaculum reached below freezing temperatures for a period of 2 consecutive days. Daily mean island hibernacula temperatures were warmer than ambient temperatures (*T*_diff_ = 1.53, CI = −1.1 to 4.15) for all months except April, and March was the only month confidence intervals overlapped zero (electronic supplementary material, figure S8). During opportunistic counts of hibernating bats within island sites, the only species present was northern myotis with a maximum count of five individuals.

## 4. Discussion

The introduction of novel pathogens can exert strong selective pressure on populations [55], and pre-existing host traits may interact with environmental conditions to promote survival from infectious disease [15,27]. While WNS has caused widespread declines in multiple bat species across North America, some populations have begun to stabilize [21,56]. Hibernating populations of northern myotis have experienced overall declines of 99% throughout much of their core range and many local colonies have been extirpated within 4 years of pathogen arrival. The probability of capturing this species on the landscape on a given night has dropped from 63% to 4% within 9 years post-WNS arrival, mirroring the severe declines of hibernating populations. However, island populations appear to be persisting at greater densities, as the capture probability declined only 6% within the same time frame. These populations on the periphery of the core range exhibit activity patterns that are facilitated by mild environmental conditions and have been documented hibernating locally in low densities, both factors that appear to slow disease progression. The coastal climate allows island bats to delay infection by remaining active on the landscape later during autumn swarm, and ultimately results in a shorter duration of hibernation compared to the mainland that is interspersed with winter activity, including foraging [57]. Our results suggest that the coastal region of this species range may serve as refugia from disease where local environmental conditions and corresponding bat activity reduce the intensity of host–pathogen interactions and thus play a key role in host population persistence.

Our comparison of pathogen prevalence among island populations suggests that Nantucket remains in the invasion phase of WNS and Long Island and Martha’s Vineyard are in the post-invasion phase (3+ years after pathogen arrival). The delayed arrival of *P. destructans* to Nantucket may be due to its distance offshore compared to the other islands resulting in relative isolation and reduced movement of bats between the island and the mainland. Our mainland data support previous evidence suggesting that northern myotis are generally extirpated from hibernacula within 3 years of *P. destructans* arrival [23]; 97% of mainland samples collected during late hibernation were from populations in the invasion phase as there were few if any individuals to sample after year 3 in these sites, and overall colony counts declined 99% by year 4. In contrast, spring sampling on the islands was conducted during April and May when individuals had emerged onto the landscape, and thus represent post-hibernation survival despite pathogen exposure up to 9 years after WNS was first detected.

Bats infected with *P. destructans* recover to a normal state quickly after emergence from hibernation, and most signs of the disease aside from visible wing damage are gone within two weeks [26]. However, island bats begin emerging in mid-March when the weather is variable and food availability is limited. The high spring prevalence we observed on the islands may result from reinfections if individuals continue roosting in hibernacula with an established environmental reservoir on unfavourable weather nights and enter a state of torpor that consequently suppresses an immune response to infection [58]. Alternatively, positive samples from bats captured on the landscape may not represent active infections but rather the presence of fungal DNA as histology is required to confirm the subcutaneous infection by *P. destructans* characteristic of the disease [59]. Spring body mass of island bats was comparable between pre- and post-WNS years, and the lack of severe wing damage suggests that individuals within these populations may not experience a degree of infection that results in functional impairment [60]. The situation on the islands is unique in that we have documented persisting northern myotis populations following disease establishment, which is a rarity in most WNS studies for this species, although it remains uncertain whether these peripheral populations occurring in refugia are self-sustainable in the long term and able to prevent total species extinction. More extensive sampling during late hibernation and spring with greater spatial and temporal coverage across the periphery of the species range could help to disentangle disease dynamics among these populations.

Reduced disease severity may be mediated by the coastal environmental conditions that result in a shorter duration of hibernation and thus allow less time for the disease to progress. Northern myotis autumn activity differs between the islands and the mainland such that island bats are active on the landscape well into November and do not appear to engage in ‘typical’ swarm behaviour at hibernacula with large congregations of allospecifics [30], resulting in significantly lower prevalence and pathogen loads prior to hibernation. In contrast, mainland populations begin swarming by the end of August upon returning to hibernacula, and high contact rates with other bats and the pathogen reservoir within these sites lead to widespread infection early in the seasonal epidemic [21,42]. The estimated duration of hibernation was up to 11 weeks shorter on the islands compared to that of the mainland (81 days versus 154 days) based on the number of days that night minimum temperatures fall below 0°C. The period between infection and mortality for WNS (70–100 days [25,61]) indicates that while most bats eventually become infected by the end of hibernation on the islands, many apparently may survive until spring when they can emerge from hibernation and clear infection [42]. While the cumulative decline in acoustic activity during autumn and subsequent increase in spring resulted in a longer period of inactivity compared to our temperature-based estimation, coverage was not spatially or temporally comprehensive and further surveys at hibernacula entrances or potential swarm sites may provide additional information to refine these dates. Although the timing of entry into and emergence from hibernation for temperate bats is relatively consistent among years it can vary with annual weather conditions [62], and warming winters due to climate change may further reduce the hibernation duration on the islands in the coming years.

The mild climate on the islands facilitated periodic activity throughout the winter months on nights with average temperatures of 5.5°C. These conditions occurred on more than 35% of winter nights, yet we only detected northern myotis on 4.5% of winter nights, indicating that our measure of winter activity may be underestimated given that we surveyed landscape sites rather than outside of hibernacula where activity may have been greater, and we had the fewest detectors operating during winter months with gaps in sampling due to detector malfunctions. Arousal episodes increase body temperature above the optimal growth range of the fungus [39], which subsequently may support immunological defences against pathogen infection [63]. In addition, WNS-affected bats increase grooming behaviour following arousals [64], which may potentially aid in the reduction of fungal loads. Recent studies provide evidence that populations of northern myotis in the southeast and along the Atlantic Coastal Plain (including the populations located on our study islands) are foraging during winter and have opportunities to drink from open water sources [57,65,66], which may provide concentrations of winter-active insects. These activities may enable bats to replenish energy stores and reduce the evaporative water loss and electrolyte imbalance that occur following disease-induced arousals [60]. While reliance on the abiotic factors that support population plasticity in marginal habitats may not have been necessary for survival prior to WNS arrival, these environmental conditions now appear to facilitate survival despite infection by reducing the intensity of host–pathogen interactions. However, the benefits of these local adaptations are not without their costs if extreme weather events (e.g. hurricanes, flooding) result in substantial mortality and a subsequent loss of genetic diversity.

Anthropogenic-mediated introductions of pathogens have caused drastic population declines and driven species to extirpation and extinction [23,67,68]. Host–pathogen interactions are not the same in all locations throughout a species range, and survival may depend on the influence of environmental variability and host traits to mediate disease outcomes. Understanding the factors driving survival during disease epidemics is critical for developing effective management strategies to support these populations, as persistence could depend on the ability of hosts to adapt, and the implementation of conservation efforts aimed at reducing impacts among affected populations. Our results suggest that the variable environmental conditions of peripheral habitats may provide refugia from disease during invasion through a reduction in the frequency or intensity of host–pathogen interactions, and consequently we expect that these outcomes balance the costly adaptations needed to survive in the periphery. However, peripheral populations may not remain viable if the delayed disease-induced declines we documented continue, and they may require additional conservation strategies to maintain or increase numbers. Management strategies could focus on actions that support survival and reproduction [69] to facilitate the evolution of resistance and tolerance [70,71]. Protecting these refugia from disturbance [72], potentially through the creation of protected artificial hibernacula, and reducing stressors other than the focal disease [70], such as habitat loss and fragmentation, may promote over-winter survival and ensure suitable habitat remains available. While environmental heterogeneity may mediate disease risk for remnant populations existing at the periphery of their range, actions supporting the ecological and evolutionary responses of hosts may be beneficial to support their future persistence.

## Data Availability

The data and code used to produce the results of this paper are available from the Dryad Digital Repository [73]. Electronic supplementary material is available online [74].
